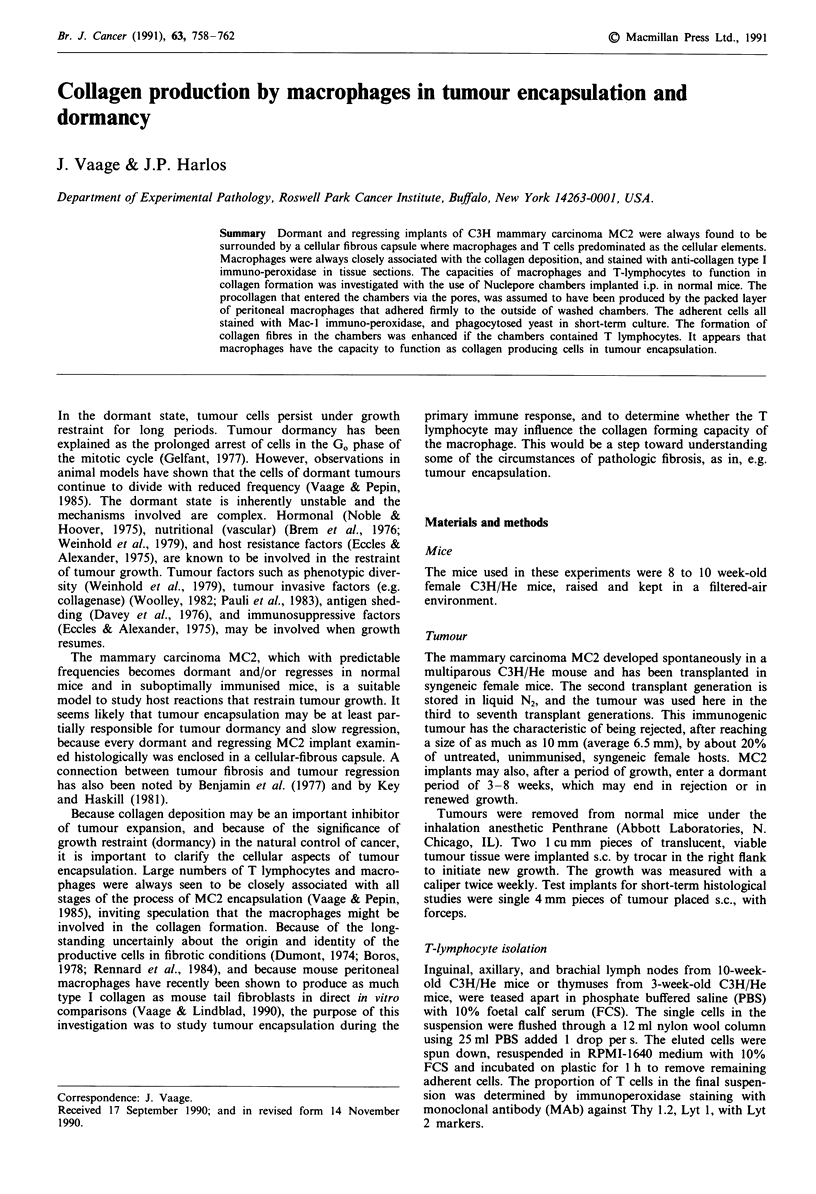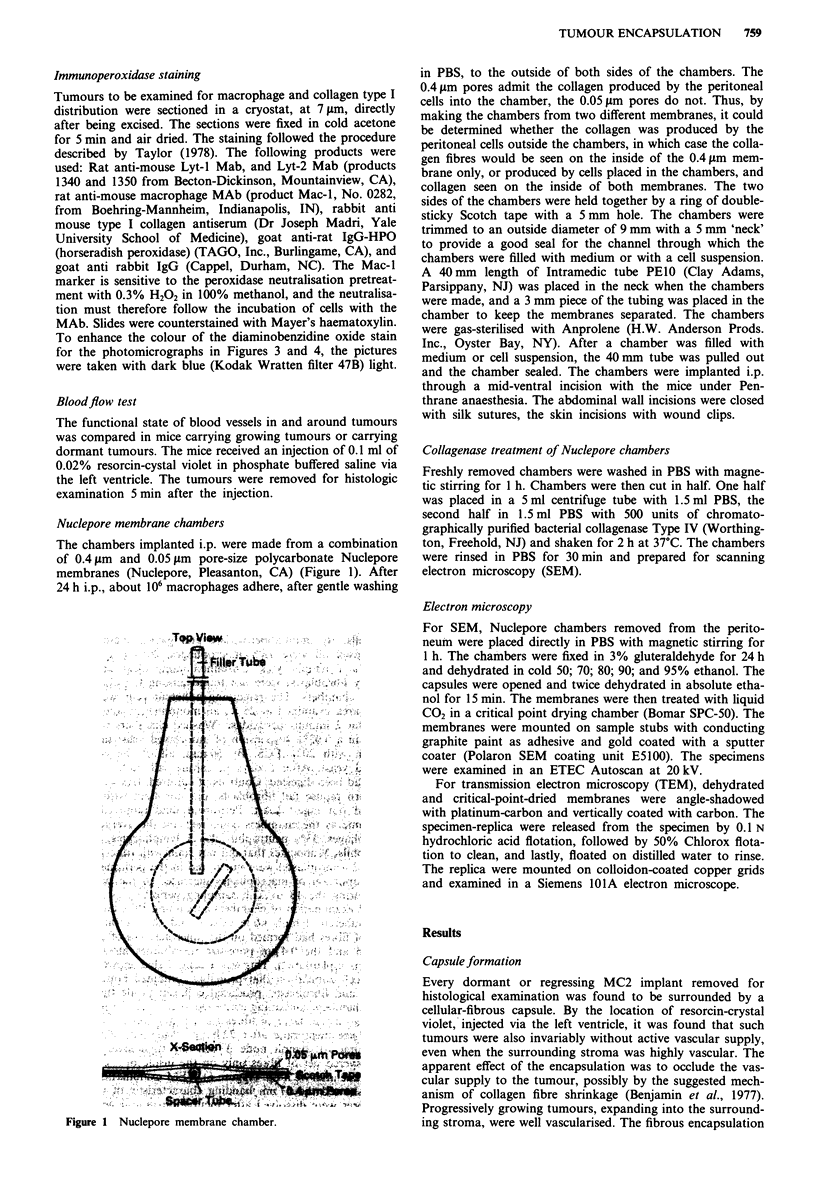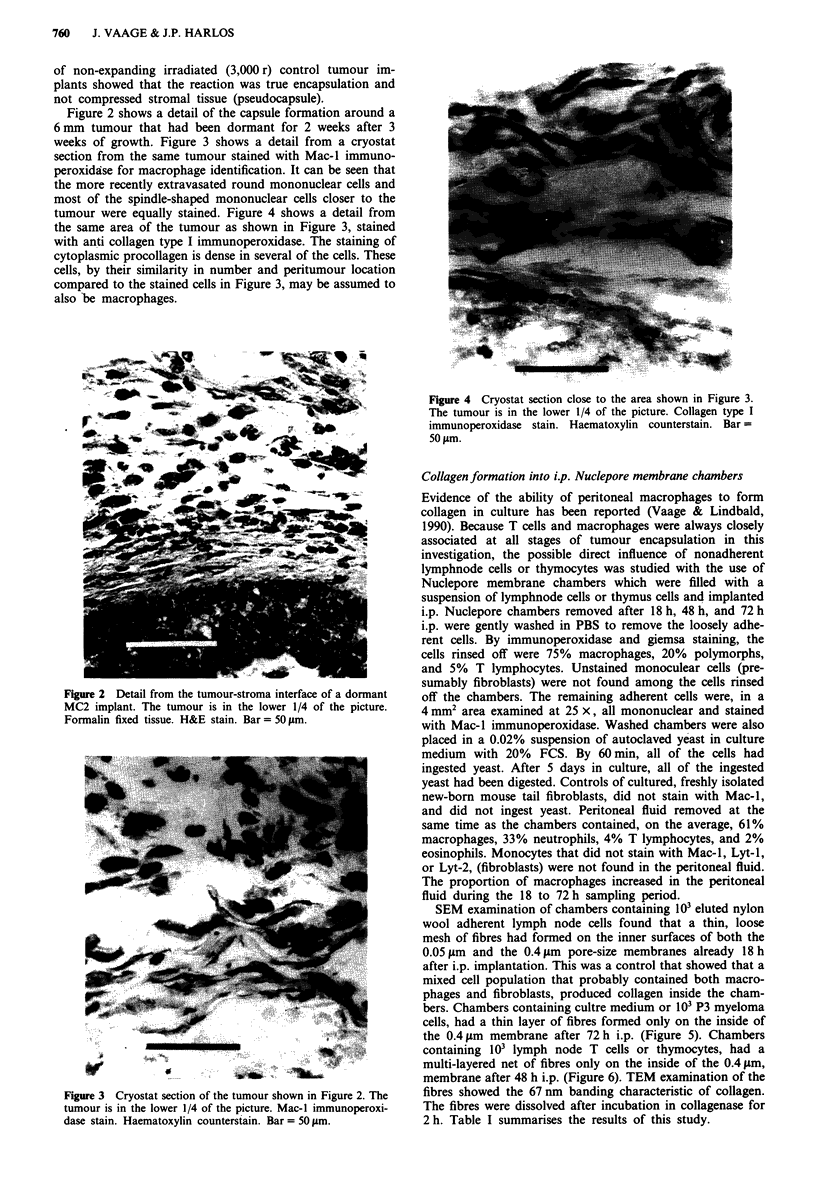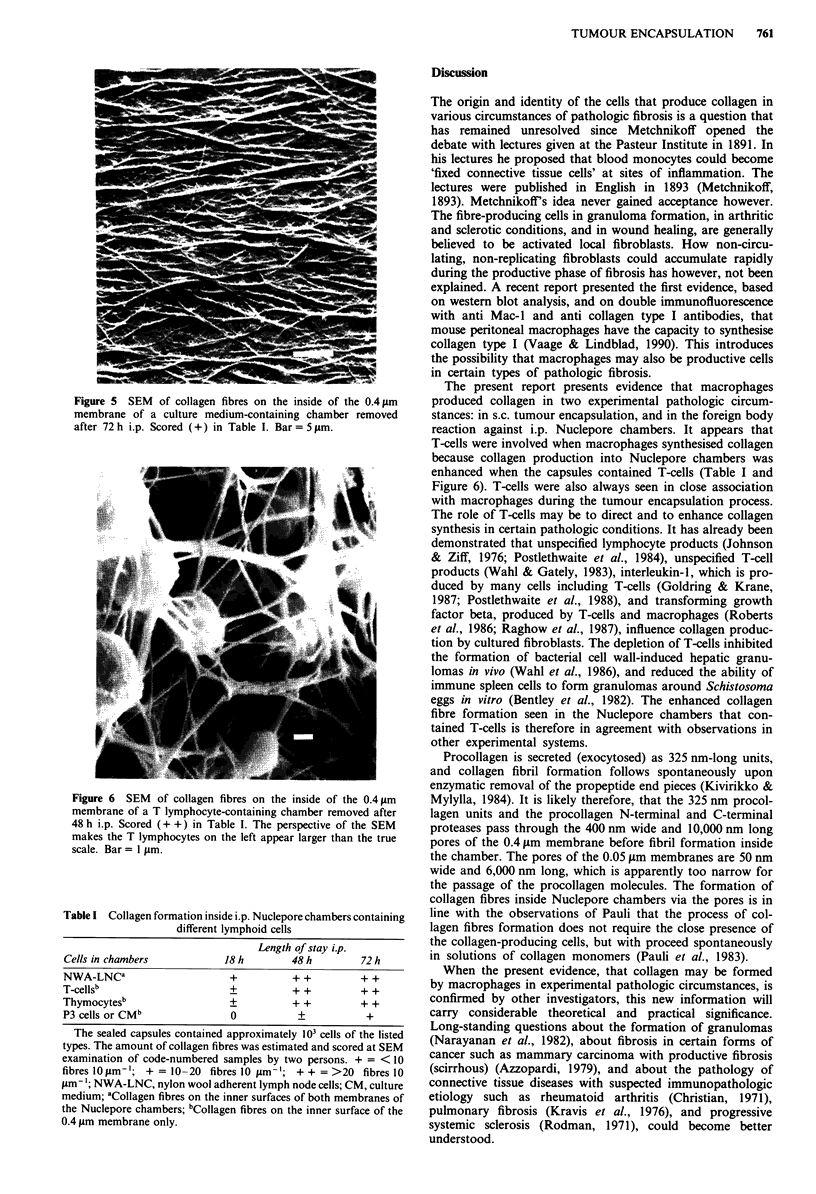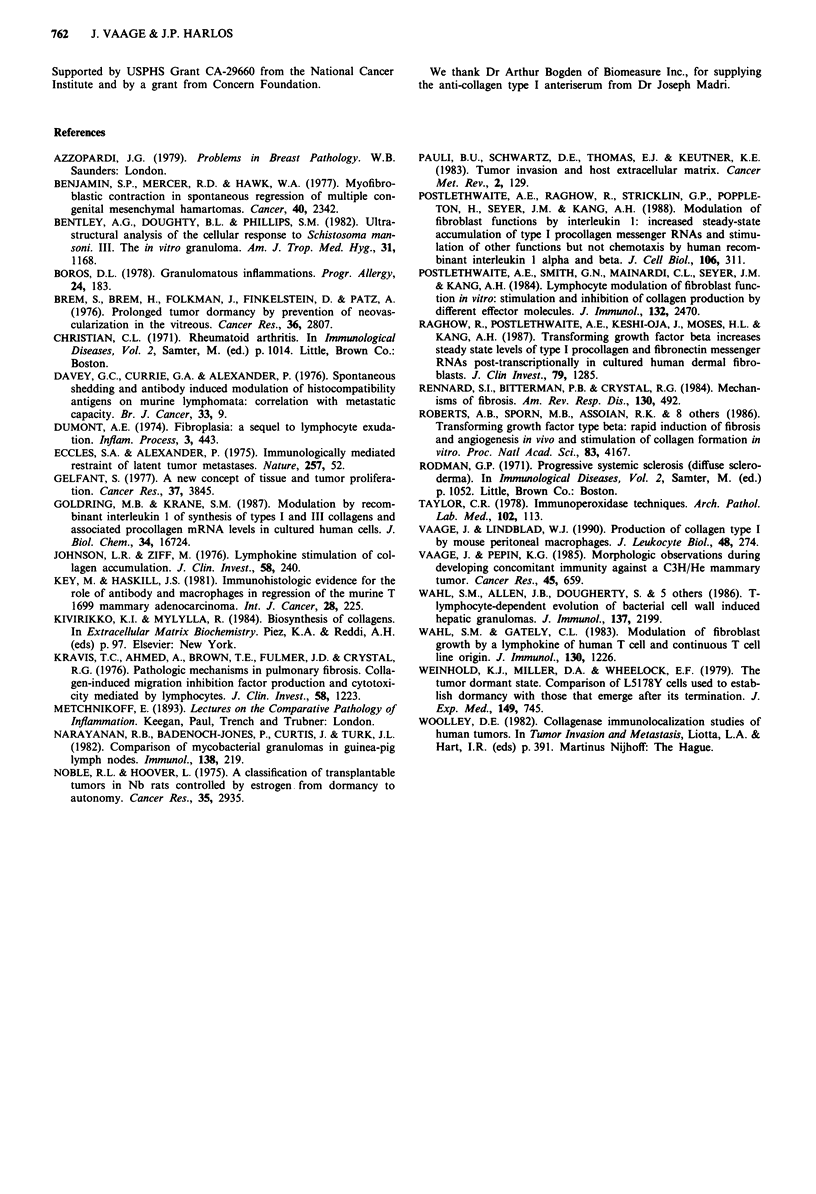# Collagen production by macrophages in tumour encapsulation and dormancy.

**DOI:** 10.1038/bjc.1991.169

**Published:** 1991-05

**Authors:** J. Vaage, J. P. Harlos

**Affiliations:** Department of Experimental Pathology, Roswell Park Cancer Institute, Buffalo, New York 14263-0001.

## Abstract

**Images:**


Br. J. Cancer (1991), 63, 758-762 © Macmillan Press Ltd., 1991

### Collagen production by macrophages in tumour encapsulation and dormancy

J. Vaage & J.P. Harlos

*Department of Experimental Pathology, Roswell Park Cancer Institute, Buffalo, New York 14263-0001, USA.*

**Summary** Dormant and regressing implants of C3H mammary carcinoma MC2 were always found to be surrounded by a cellular fibrous capsule where macrophages and T cells predominated as the cellular elements. Macrophages were always closely associated with the collagen deposition, and stained with anti-collagen type I immuno-peroxidase in tissue sections. The capacities of macrophages and T-lymphocytes to function in collagen formation was investigated with the use of Nuclepore chambers implanted i.p. in normal mice. The procollagen that entered the chambers via the pores, was assumed to have been produced by the packed layer of peritoneal macrophages that adhered firmly to the outside of washed chambers. The adherent cells all stained with Mac-1 immuno-peroxidase, and phagocytosed yeast in short-term culture. The formation of collagen fibres in the chambers was enhanced if the chambers contained T lymphocytes. It appears that macrophages have the capacity to function as collagen producing cells in tumour encapsulation.

In the dormant state, tumour cells persist under growth restraint for long periods. Tumour dormancy has been explained as the prolonged arrest of cells in the $G_0$ phase of the mitotic cycle (Gelfant, 1977). However, observations in animal models have shown that the cells of dormant tumours continue to divide with reduced frequency (Vaage & Pepin, 1985). The dormant state is inherently unstable and the mechanisms involved are complex. Hormonal (Noble & Hoover, 1975), nutritional (vascular) (Brem *et al.*, 1976; Weinhold *et al.*, 1979), and host resistance factors (Eccles & Alexander, 1975), are known to be involved in the restraint of tumour growth. Tumour factors such as phenotypic diversity (Weinhold *et al.*, 1979), tumour invasive factors (e.g. collagenase) (Woolley, 1982; Pauli *et al.*, 1983), antigen shedding (Davey *et al.*, 1976), and immunosuppressive factors (Eccles & Alexander, 1975), may be involved when growth resumes.

The mammary carcinoma MC2, which with predictable frequencies becomes dormant and/or regresses in normal mice and in suboptimally immunised mice, is a suitable model to study host reactions that restrain tumour growth. It seems likely that tumour encapsulation may be at least partially responsible for tumour dormancy and slow regression, because every dormant and regressing MC2 implant examined histologically was enclosed in a cellular-fibrous capsule. A connection between tumour fibrosis and tumour regression has also been noted by Benjamin *et al.* (1977) and by Key and Haskill (1981).

Because collagen deposition may be an important inhibitor of tumour expansion, and because of the significance of growth restraint (dormancy) in the natural control of cancer, it is important to clarify the cellular aspects of tumour encapsulation. Large numbers of T lymphocytes and macrophages were always seen to be closely associated with all stages of the process of MC2 encapsulation (Vaage & Pepin, 1985), inviting speculation that the macrophages might be involved in the collagen formation. Because of the longstanding uncertainly about the origin and identity of the productive cells in fibrotic conditions (Dumont, 1974; Boros, 1978; Rennard *et al.*, 1984), and because mouse peritoneal macrophages have recently been shown to produce as much type I collagen as mouse tail fibroblasts in direct *in vitro* comparisons (Vaage & Lindblad, 1990), the purpose of this investigation was to study tumour encapsulation during the primary immune response, and to determine whether the T lymphocyte may influence the collagen forming capacity of the macrophage. This would be a step toward understanding some of the circumstances of pathologic fibrosis, as in, e.g. tumour encapsulation.

### Materials and methods

### *Mice*

The mice used in these experiments were 8 to 10 week-old female C3H/He mice, raised and kept in a filtered-air environment.

### *Tumour*

The mammary carcinoma MC2 developed spontaneously in a multiparous C3H/He mouse and has been transplanted in syngeneic female mice. The second transplant generation is stored in liquid $N_2$, and the tumour was used here in the third to seventh transplant generations. This immunogenic tumour has the characteristic of being rejected, after reaching a size of as much as 10 mm (average 6.5 mm), by about 20% of untreated, unimmunised, syngeneic female hosts. MC2 implants may also, after a period of growth, enter a dormant period of 3-8 weeks, which may end in rejection or in renewed growth.

Tumours were removed from normal mice under the inhalation anesthetic Penthrane (Abbott Laboratories, N. Chicago, IL). Two 1 cu mm pieces of translucent, viable tumour tissue were implanted s.c. by trocar in the right flank to initiate new growth. The growth was measured with a caliper twice weekly. Test implants for short-term histological studies were single 4 mm pieces of tumour placed s.c., with forceps.

### *T-lymphocyte isolation*

Inguinal, axillary, and brachial lymph nodes from 10-week-old C3H/He mice or thymuses from 3-week-old C3H/He mice, were teased apart in phosphate buffered saline (PBS) with 10% foetal calf serum (FCS). The single cells in the suspension were flushed through a 12 ml nylon wool column using 25 ml PBS added 1 drop per s. The eluted cells were spun down, resuspended in RPMI-1640 medium with 10% FCS and incubated on plastic for 1 h to remove remaining adherent cells. The proportion of T cells in the final suspension was determined by immunoperoxidase staining with monoclonal antibody (MAb) against Thy 1.2, Lyt 1, with Lyt 2 markers.

Correspondence: J. Vaage.
Received 17 September 1990; and in revised form 14 November 1990.

*Immunoperoxidase staining*

Tumours to be examined for macrophage and collagen type I distribution were sectioned in a cryostat, at 7 µm, directly after being excised. The sections were fixed in cold acetone for 5 min and air dried. The staining followed the procedure described by Taylor (1978). The following products were used: Rat anti-mouse Lyt-1 Mab, and Lyt-2 Mab (products 1340 and 1350 from Becton-Dickinson, Mountainview, CA), rat anti-mouse macrophage MAb (product Mac-1, No. 0282, from Boehring-Mannheim, Indianapolis, IN), rabbit anti mouse type I collagen antiserum (Dr Joseph Madri, Yale University School of Medicine), goat anti-rat IgG-HPO (horseradish peroxidase) (TAGO, Inc., Burlingame, CA), and goat anti rabbit IgG (Cappel, Durham, NC). The Mac-1 marker is sensitive to the peroxidase neutralisation pretreatment with 0.3% $H_2O_2$ in 100% methanol, and the neutralisation must therefore follow the incubation of cells with the MAb. Slides were counterstained with Mayer's haematoxylin. To enhance the colour of the diaminobenzidine oxide stain for the photomicrographs in Figures 3 and 4, the pictures were taken with dark blue (Kodak Wratten filter 47B) light.

*Blood flow test*

The functional state of blood vessels in and around tumours was compared in mice carrying growing tumours or carrying dormant tumours. The mice received an injection of 0.1 ml of 0.02% resorcin-cystal violet in phosphate buffered saline via the left ventricle. The tumours were removed for histologic examination 5 min after the injection.

*Nuclepore membrane chambers*

The chambers implanted i.p. were made from a combination of 0.4 µm and 0.05 µm pore-size polycarbonate Nuclepore membranes (Nuclepore, Pleasanton, CA) (Figure 1). After 24 h i.p., about $10^6$ macrophages adhere, after gentle washing in PBS, to the outside of both sides of the chambers. The 0.4 µm pores admit the collagen produced by the peritoneal cells into the chamber, the 0.05 µm pores do not. Thus, by making the chambers from two different membranes, it could be determined whether the collagen was produced by the peritoneal cells outside the chambers, in which case the collagen fibres would be seen on the inside of the 0.4 µm membrane only, or produced by cells placed in the chambers, and collagen seen on the inside of both membranes. The two sides of the chambers were held together by a ring of double-sticky Scotch tape with a 5 mm hole. The chambers were trimmed to an outside diameter of 9 mm with a 5 mm 'neck' to provide a good seal for the channel through which the chambers were filled with medium or with a cell suspension. A 40 mm length of Intramedic tube PE10 (Clay Adams, Parsippany, NJ) was placed in the neck when the chambers were made, and a 3 mm piece of the tubing was placed in the chamber to keep the membranes separated. The chambers were gas-sterilised with Anprolene (H.W. Anderson Prods. Inc., Oyster Bay, NY). After a chamber was filled with medium or cell suspension, the 40 mm tube was pulled out and the chamber sealed. The chambers were implanted i.p. through a mid-ventral incision with the mice under Penthrane anaesthesia. The abdominal wall incisions were closed with silk sutures, the skin incisions with wound clips.

**Figure 1** Nuclepore membrane chamber.

*Collagenase treatment of Nuclepore chambers*

Freshly removed chambers were washed in PBS with magnetic stirring for 1 h. Chambers were then cut in half. One half was placed in a 5 ml centrifuge tube with 1.5 ml PBS, the second half in 1.5 ml PBS with 500 units of chromatographically purified bacterial collagenase Type IV (Worthington, Freehold, NJ) and shaken for 2 h at 37°C. The chambers were rinsed in PBS for 30 min and prepared for scanning electron microscopy (SEM).

*Electron microscopy*

For SEM, Nuclepore chambers removed from the peritoneum were placed directly in PBS with magnetic stirring for 1 h. The chambers were fixed in 3% gluteraldehyde for 24 h and dehydrated in cold 50; 70; 80; 90; and 95% ethanol. The capsules were opened and twice dehydrated in absolute ethanol for 15 min. The membranes were then treated with liquid $CO_2$ in a critical point drying chamber (Bomar SPC-50). The membranes were mounted on sample stubs with conducting graphite paint as adhesive and gold coated with a sputter coater (Polaron SEM coating unit E5100). The specimens were examined in an ETEC Autoscan at 20 kV.

For transmission electron microscopy (TEM), dehydrated and critical-point-dried membranes were angle-shadowed with platinum-carbon and vertically coated with carbon. The specimen-replica were released from the specimen by 0.1 N hydrochloric acid flotation, followed by 50% Chlorox flotation to clean, and lastly, floated on distilled water to rinse. The replica were mounted on colloidon-coated copper grids and examined in a Siemens 101A electron microscope.

### Results

*Capsule formation*

Every dormant or regressing MC2 implant removed for histological examination was found to be surrounded by a cellular-fibrous capsule. By the location of resorcin-crystal violet, injected via the left ventricle, it was found that such tumours were also invariably without active vascular supply, even when the surrounding stroma was highly vascular. The apparent effect of the encapsulation was to occlude the vascular supply to the tumour, possibly by the suggested mechanism of collagen fibre shrinkage (Benjamin *et al.*, 1977). Progressively growing tumours, expanding into the surrounding stroma, were well vascularised. The fibrous encapsulation of non-expanding irradiated (3,000 r) control tumour implants showed that the reaction was true encapsulation and not compressed stromal tissue (pseudocapsule).

Figure 2 shows a detail of the capsule formation around a 6 mm tumour that had been dormant for 2 weeks after 3 weeks of growth. Figure 3 shows a detail from a cryostat section from the same tumour stained with Mac-1 immunoperoxidase for macrophage identification. It can be seen that the more recently extravasated round mononuclear cells and most of the spindle-shaped mononuclear cells closer to the tumour were equally stained. Figure 4 shows a detail from the same area of the tumour as shown in Figure 3, stained with anti collagen type I immunoperoxidase. The staining of cytoplasmic procollagen is dense in several of the cells. These cells, by their similarity in number and peritumour location compared to the stained cells in Figure 3, may be assumed to also be macrophages.

**Figure 2** Detail from the tumour-stroma interface of a dormant MC2 implant. The tumour is in the lower 1/4 of the picture. Formalin fixed tissue. H&E stain. Bar = 50 $\mu$m.

**Figure 3** Cryostat section of the tumour shown in Figure 2. The tumour is in the lower 1/4 of the picture. Mac-1 immunoperoxidase stain. Haematoxylin counterstain. Bar = 50 $\mu$m.

**Figure 4** Cryostat section close to the area shown in Figure 3. The tumour is in the lower 1/4 of the picture. Collagen type I immunoperoxidase stain. Haematoxylin counterstain. Bar = 50 $\mu$m.

*Collagen formation into i.p. Nuclepore membrane chambers*

Evidence of the ability of peritoneal macrophages to form collagen in culture has been reported (Vaage & Lindbald, 1990). Because T cells and macrophages were always closely associated at all stages of tumour encapsulation in this investigation, the possible direct influence of nonadherent lymphnode cells or thymocytes was studied with the use of Nuclepore membrane chambers which were filled with a suspension of lymphnode cells or thymus cells and implanted i.p. Nuclepore chambers removed after 18 h, 48 h, and 72 h i.p. were gently washed in PBS to remove the loosely adherent cells. By immunoperoxidase and giemsa staining, the cells rinsed off were 75% macrophages, 20% polymorphs, and 5% T lymphocytes. Unstained monoculear cells (presumably fibroblasts) were not found among the cells rinsed off the chambers. The remaining adherent cells were, in a 4 mm$^2$ area examined at 25 ×, all mononuclear and stained with Mac-1 immunoperoxidase. Washed chambers were also placed in a 0.02% suspension of autoclaved yeast in culture medium with 20% FCS. By 60 min, all of the cells had ingested yeast. After 5 days in culture, all of the ingested yeast had been digested. Controls of cultured, freshly isolated new-born mouse tail fibroblasts, did not stain with Mac-1, and did not ingest yeast. Peritoneal fluid removed at the same time as the chambers contained, on the average, 61% macrophages, 33% neutrophils, 4% T lymphocytes, and 2% eosinophils. Monocytes that did not stain with Mac-1, Lyt-1, or Lyt-2, (fibroblasts) were not found in the peritoneal fluid. The proportion of macrophages increased in the peritoneal fluid during the 18 to 72 h sampling period.

SEM examination of chambers containing $10^3$ eluted nylon wool adherent lymph node cells found that a thin, loose mesh of fibres had formed on the inner surfaces of both the 0.05 $\mu$m and the 0.4 $\mu$m pore-size membranes already 18 h after i.p. implantation. This was a control that showed that a mixed cell population that probably contained both macrophages and fibroblasts, produced collagen inside the chambers. Chambers containing cultre medium or $10^3$ P3 myeloma cells, had a thin layer of fibres formed only on the inside of the 0.4 $\mu$m membrane after 72 h i.p. (Figure 5). Chambers containing $10^3$ lymph node T cells or thymocytes, had a multi-layered net of fibres only on the inside of the 0.4 $\mu$m, membrane after 48 h i.p. (Figure 6). TEM examination of the fibres showed the 67 nm banding characteristic of collagen. The fibres were dissolved after incubation in collagenase for 2 h. Table I summarises the results of this study.

**Figure 5** SEM of collagen fibres on the inside of the 0.4 μm membrane of a culture medium-containing chamber removed after 72 h i.p. Scored (+) in Table I. Bar = 5 μm.

**Figure 6** SEM of collagen fibres on the inside of the 0.4 μm membrane of a T lymphocyte-containing chamber removed after 48 h i.p. Scored (+ +) in Table I. The perspective of the SEM makes the T lymphocytes on the left appear larger than the true scale. Bar = 1 μm.

**Table I** Collagen formation inside i.p. Nuclepore chambers containing different lymphoid cells

| | *Length of stay i.p.* | | |
|---|---|---|---|
| *Cells in chambers* | *18 h* | *48 h* | *72 h* |
| NWA-LNC[a] | + | + + | + + |
| T-cells[b] | ± | + + | + + |
| Thymocytes[b] | ± | + + | + + |
| P3 cells or CM[b] | 0 | ± | + |

The sealed capsules contained approximately $10^3$ cells of the listed types. The amount of collagen fibres was estimated and scored at SEM examination of code-numbered samples by two persons. + = <10 fibres 10 $\mu m^{-1}$; + = 10–20 fibres 10 $\mu m^{-1}$; + + = >20 fibres 10 $\mu m^{-1}$; NWA-LNC, nylon wool adherent lymph node cells; CM, culture medium; [a]Collagen fibres on the inner surfaces of both membranes of the Nuclepore chambers; [b]Collagen fibres on the inner surface of the 0.4 μm membrane only.

### Discussion

The origin and identity of the cells that produce collagen in various circumstances of pathologic fibrosis is a question that has remained unresolved since Metchnikoff opened the debate with lectures given at the Pasteur Institute in 1891. In his lectures he proposed that blood monocytes could become 'fixed connective tissue cells' at sites of inflammation. The lectures were published in English in 1893 (Metchnikoff, 1893). Metchnikoff's idea never gained acceptance however. The fibre-producing cells in granuloma formation, in arthritic and sclerotic conditions, and in wound healing, are generally believed to be activated local fibroblasts. How non-circulating, non-replicating fibroblasts could accumulate rapidly during the productive phase of fibrosis has however, not been explained. A recent report presented the first evidence, based on western blot analysis, and on double immunofluorescence with anti Mac-1 and anti collagen type I antibodies, that mouse peritoneal macrophages have the capacity to synthesise collagen type I (Vaage & Lindblad, 1990). This introduces the possibility that macrophages may also be productive cells in certain types of pathologic fibrosis.

The present report presents evidence that macrophages produced collagen in two experimental pathologic circumstances: in s.c. tumour encapsulation, and in the foreign body reaction against i.p. Nuclepore chambers. It appears that T-cells were involved when macrophages synthesised collagen because collagen production into Nuclepore chambers was enhanced when the capsules contained T-cells (Table I and Figure 6). T-cells were also always seen in close association with macrophages during the tumour encapsulation process. The role of T-cells may be to direct and to enhance collagen synthesis in certain pathologic conditions. It has already been demonstrated that unspecified lymphocyte products (Johnson & Ziff, 1976; Postlethwaite *et al.*, 1984), unspecified T-cell products (Wahl & Gately, 1983), interleukin-1, which is produced by many cells including T-cells (Goldring & Krane, 1987; Postlethwaite *et al.*, 1988), and transforming growth factor beta, produced by T-cells and macrophages (Roberts *et al.*, 1986; Raghow *et al.*, 1987), influence collagen production by cultured fibroblasts. The depletion of T-cells inhibited the formation of bacterial cell wall-induced hepatic granulomas *in vivo* (Wahl *et al.*, 1986), and reduced the ability of immune spleen cells to form granulomas around *Schistosoma* eggs *in vitro* (Bentley *et al.*, 1982). The enhanced collagen fibre formation seen in the Nuclepore chambers that contained T-cells is therefore in agreement with observations in other experimental systems.

Procollagen is secreted (exocytosed) as 325 nm-long units, and collagen fibril formation follows spontaneously upon enzymatic removal of the propeptide end pieces (Kivirikko & Mylylla, 1984). It is likely therefore, that the 325 nm procollagen units and the procollagen N-terminal and C-terminal proteases pass through the 400 nm wide and 10,000 nm long pores of the 0.4 μm membrane before fibril formation inside the chamber. The pores of the 0.05 μm membranes are 50 nm wide and 6,000 nm long, which is apparently too narrow for the passage of the procollagen molecules. The formation of collagen fibres inside Nuclepore chambers via the pores is in line with the observations of Pauli that the process of collagen fibres formation does not require the close presence of the collagen-producing cells, but with proceed spontaneously in solutions of collagen monomers (Pauli *et al.*, 1983).

When the present evidence, that collagen may be formed by macrophages in experimental pathologic circumstances, is confirmed by other investigators, this new information will carry considerable theoretical and practical significance. Long-standing questions about the formation of granulomas (Narayanan *et al.*, 1982), about fibrosis in certain forms of cancer such as mammary carcinoma with productive fibrosis (scirrhous) (Azzopardi, 1979), and about the pathology of connective tissue diseases with suspected immunopathologic etiology such as rheumatoid arthritis (Christian, 1971), pulmonary fibrosis (Kravis *et al.*, 1976), and progressive systemic sclerosis (Rodman, 1971), could become better understood.

Supported by USPHS Grant CA-29660 from the National Cancer Institute and by a grant from Concern Foundation.

We thank Dr Arthur Bogden of Biomeasure Inc., for supplying the anti-collagen type I anteriserum from Dr Joseph Madri.

### References

AZZOPARDI, J.G. (1979). *Problems in Breast Pathology*. W.B. Saunders: London.

BENJAMIN, S.P., MERCER, R.D. & HAWK, W.A. (1977). Myofibroblastic contraction in spontaneous regression of multiple congenital mesenchymal hamartomas. *Cancer*, **40**, 2342.

BENTLEY, A.G., DOUGHTY, B.L. & PHILLIPS, S.M. (1982). Ultrastructural analysis of the cellular response to *Schistosoma mansoni*. III. The *in vitro* granuloma. *Am. J. Trop. Med. Hyg.*, **31**, 1168.

BOROS, D.L. (1978). Granulomatous inflammations. *Progr. Allergy*, **24**, 183.

BREM, S., BREM, H., FOLKMAN, J., FINKELSTEIN, D. & PATZ, A. (1976). Prolonged tumor dormancy by prevention of neovascularization in the vitreous. *Cancer Res.*, **36**, 2807.

CHRISTIAN, C.L. (1971). Rheumatoid arthritis. In *Immunological Diseases, Vol. 2*, Samter, M. (ed.) p. 1014. Little, Brown Co.: Boston.

DAVEY, G.C., CURRIE, G.A. & ALEXANDER, P. (1976). Spontaneous shedding and antibody induced modulation of histocompatibility antigens on murine lymphomata: correlation with metastatic capacity. *Br. J. Cancer*, **33**, 9.

DUMONT, A.E. (1974). Fibroplasia: a sequel to lymphocyte exudation. *Inflam. Process*, **3**, 443.

ECCLES, S.A. & ALEXANDER, P. (1975). Immunologically mediated restraint of latent tumor metastases. *Nature*, **257**, 52.

GELFANT, S. (1977). A new concept of tissue and tumor proliferation. *Cancer Res.*, **37**, 3845.

GOLDRING, M.B. & KRANE, S.M. (1987). Modulation by recombinant interleukin 1 of synthesis of types I and III collagens and associated procollagen mRNA levels in cultured human cells. *J. Biol. Chem.*, **34**, 16724.

JOHNSON, L.R. & ZIFF, M. (1976). Lymphokine stimulation of collagen accumulation. *J. Clin. Invest.*, **58**, 240.

KEY, M. & HASKILL, J.S. (1981). Immunohistologic evidence for the role of antibody and macrophages in regression of the murine T 1699 mammary adenocarcinoma. *Int. J. Cancer*, **28**, 225.

KIVIRIKKO, K.I. & MYLYLLA, R. (1984). Biosynthesis of collagens. In *Extracellular Matrix Biochemistry*. Piez, K.A. & Reddi, A.H. (eds) p. 97. Elsevier: New York.

KRAVIS, T.C., AHMED, A., BROWN, T.E., FULMER, J.D. & CRYSTAL, R.G. (1976). Pathologic mechanisms in pulmonary fibrosis. Collagen-induced migration inhibition factor production and cytotoxicity mediated by lymphocytes. *J. Clin. Invest.*, **58**, 1223.

METCHNIKOFF, E. (1893). *Lectures on the Comparative Pathology of Inflammation*. Keegan, Paul, Trench and Trubner: London.

NARAYANAN, R.B., BADENOCH-JONES, P., CURTIS, J. & TURK, J.L. (1982). Comparison of mycobacterial granulomas in guinea-pig lymph nodes. *Immunol.*, **138**, 219.

NOBLE, R.L. & HOOVER, L. (1975). A classification of transplantable tumors in Nb rats controlled by estrogen from dormancy to autonomy. *Cancer Res.*, **35**, 2935.

PAULI, B.U., SCHWARTZ, D.E., THOMAS, E.J. & KEUTNER, K.E. (1983). Tumor invasion and host extracellular matrix. *Cancer Met. Rev.*, **2**, 129.

POSTLETHWAITE, A.E., RAGHOW, R., STRICKLIN, G.P., POPPLETON, H., SEYER, J.M. & KANG, A.H. (1988). Modulation of fibroblast functions by interleukin 1: increased steady-state accumulation of type I procollagen messenger RNAs and stimulation of other functions but not chemotaxis by human recombinant interleukin 1 alpha and beta. *J. Cell Biol.*, **106**, 311.

POSTLETHWAITE, A.E., SMITH, G.N., MAINARDI, C.L., SEYER, J.M. & KANG, A.H. (1984). Lymphocyte modulation of fibroblast function *in vitro*: stimulation and inhibition of collagen production by different effector molecules. *J. Immunol.*, **132**, 2470.

RAGHOW, R., POSTLETHWAITE, A.E., KESHI-OJA, J., MOSES, H.L. & KANG, A.H. (1987). Transforming growth factor beta increases steady state levels of type I procollagen and fibronectin messenger RNAs post-transcriptionally in cultured human dermal fibroblasts. *J. Clin Invest.*, **79**, 1285.

RENNARD, S.I., BITTERMAN, P.B. & CRYSTAL, R.G. (1984). Mechanisms of fibrosis. *Am. Rev. Resp. Dis.*, **130**, 492.

ROBERTS, A.B., SPORN, M.B., ASSOIAN, R.K. & 8 others (1986). Transforming growth factor type beta: rapid induction of fibrosis and angiogenesis *in vivo* and stimulation of collagen formation *in vitro*. *Proc. Natl Acad. Sci.*, **83**, 4167.

RODMAN, G.P. (1971). Progressive systemic sclerosis (diffuse scleroderma). In *Immunological Diseases, Vol. 2*, Samter, M. (ed.) p. 1052. Little, Brown Co.: Boston.

TAYLOR, C.R. (1978). Immunoperoxidase techniques. *Arch. Pathol. Lab. Med.*, **102**, 113.

VAAGE, J. & LINDBLAD, W.J. (1990). Production of collagen type I by mouse peritoneal macrophages. *J. Leukocyte Biol.*, **48**, 274.

VAAGE, J. & PEPIN, K.G. (1985). Morphologic observations during developing concomitant immunity against a C3H/He mammary tumor. *Cancer Res.*, **45**, 659.

WAHL, S.M., ALLEN, J.B., DOUGHERTY, S. & 5 others (1986). T-lymphocyte-dependent evolution of bacterial cell wall induced hepatic granulomas. *J. Immunol.*, **137**, 2199.

WAHL, S.M. & GATELY, C.L. (1983). Modulation of fibroblast growth by a lymphokine of human T cell and continuous T cell line origin. *J. Immunol.*, **130**, 1226.

WEINHOLD, K.J., MILLER, D.A. & WHEELOCK, E.F. (1979). The tumor dormant state. Comparison of L5178Y cells used to establish dormancy with those that emerge after its termination. *J. Exp. Med.*, **149**, 745.

WOOLLEY, D.E. (1982). Collagenase immunolocalization studies of human tumors. In *Tumor Invasion and Metastasis*, Liotta, L.A. & Hart, I.R. (eds) p. 391. Martinus Nijhoff: The Hague.